# Single photon detector with high polarization sensitivity

**DOI:** 10.1038/srep09616

**Published:** 2015-04-15

**Authors:** Qi Guo, Hao Li, LiXing You, WeiJun Zhang, Lu Zhang, Zhen Wang, XiaoMing Xie, Ming Qi

**Affiliations:** 1 State Key Laboratory of Functional Materials for Informatics, Shanghai Institute of Microsystem and Information Technology (SIMIT), Chinese Academy of Sciences, 865 Changning Rd., Shanghai 200050, China

## Abstract

Polarization is one of the key parameters of light. Most optical detectors are intensity detectors that are insensitive to the polarization of light. A superconducting nanowire single photon detector (SNSPD) is naturally sensitive to polarization due to its nanowire structure. Previous studies focused on producing a polarization-insensitive SNSPD. In this study, by adjusting the width and pitch of the nanowire, we systematically investigate the preparation of an SNSPD with high polarization sensitivity. Subsequently, an SNSPD with a system detection efficiency of 12% and a polarization extinction ratio of 22 was successfully prepared.

Polarization, together with amplitude, phase and frequency or wavelength, are the four fundamental properties of light. Understanding and utilizing polarization could greatly expand and enrich optical applications. Numerous applications based on the detection of polarization have been explored, such as polarization-coding quantum key distribution^1^, biomedical applications^2^,^3^, remote sensing^4^, and polarization imaging^5^. Nevertheless, most conventional optical detectors are intensity detectors, which are insensitive to the polarization of light. To achieve polarization-sensitive detection, an additional polarizing filter or stand-alone polarizer is necessary before the optical detector^6^. Advanced ultrasensitive optical detection has approached the epoch of single photon detection. However, there are no reports on semiconducting single photon detectors (SPDs) with polarization sensitivity to the best of our knowledge.

In the last decade, a novel SPD based on a superconducting meandered nanowire was demonstrated to exhibit better performance (high detection efficiency, low dark count rate, high repetition rate, and small timing jitter) beyond that of the traditional semiconducting SPDs for near infrared wavelengths^7^,^8^,^9^,^10^,^11^. The detection mechanism of the superconducting nanowire single photon detector (SNSPD) is the generation and disappearance of a photon-induced resistive domain across the superconducting nanowire upon absorptance of a photon^7^. Due to its unique anisotropic geometric structure, an SNSPD is naturally polarization sensitive^12^. However, most previous studies focused on producing a polarization-insensitive SNSPD^8^,^12^,^13^,^14^.

In this paper, we present our recent work on fabricating an SPD with high polarization sensitivity. We systematically discuss the relationships between the polarization extinction ratio (PER) and SNSPD geometric parameters (width, pitch, etc.). SNSPDs with high PER are fabricated, and a detection efficiency (DE) of 12% and PER of 22 are successfully attained.

## Results

### Model and analysis

The polarization dependence of SNSPDs originates from their anisotropic meandered nanowire geometry. For a transverse magnetic (TM) wave with the electrical field perpendicular to the nanowire, the magnetic field intensity is uniform across the wire; for a transverse electric (TE) wave with the magnetic field perpendicular to the nanowire, the electrical field intensity is uniform across the wire. When the detector is illuminated from either the top or the bottom through the substrate by a TM wave, the generated charges at the edge of the nanowire result in discontinuities of the electric field, which screen the nanowire and reduce the electric field intensity inside of the nanowire leading to decreased absorptance. However, for a TE wave, no discontinuities appear at the edge of the nanowire, and the electric field is relatively evenly distributed across the wire, which results in a higher absorptance. Therefore, the absorptance difference of the nanowire for TM and TE waves leads to the polarization sensitivity of the SNSPD. In general, to achieve high polarization sensitivity, the nanowire should be narrow and with a smaller filling ratio.

**Figure 1:**
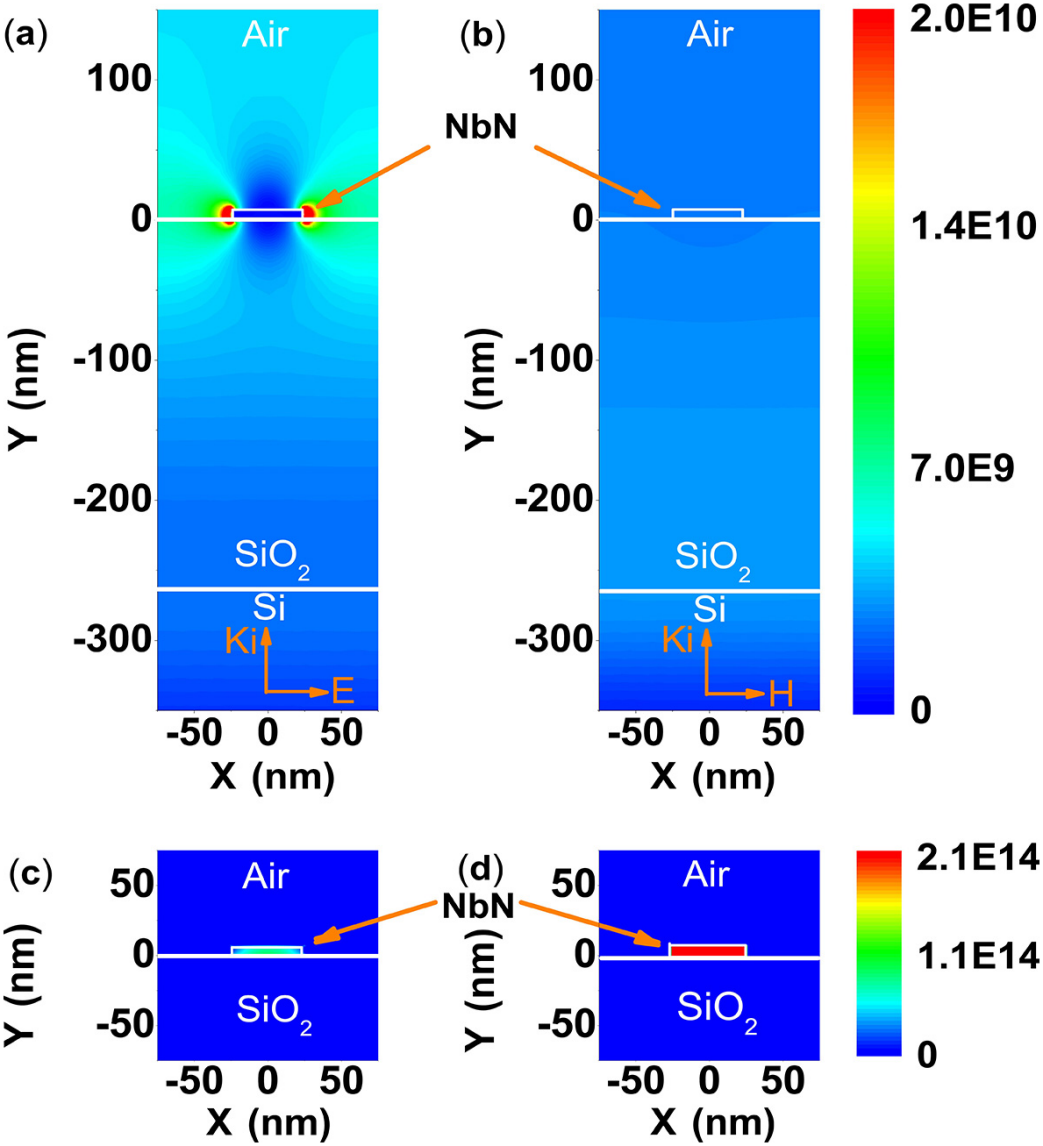
Simulation results of SNSPD. (a) & (b) Electric field intensity distribution when TM (a) and TE (b) waves are incident on the nanowire from the back of the substrate. (c) & (d) Electromagnetic power loss density distribution for TM (c) and TE (d) waves.

To quantitatively investigate the polarization sensitivity of the SNSPD, we performed numerical simulations using the finite element method (FEM). The physical picture is the same for both front- and back-side illumination. Backside illumination is selected here for the possibility of integrating an optical cavity on the top in the future. With Comsol Multiphysics, under RF module, a model is set up as shown in Figs. 1(a) and (b) for one unit cell of the grating, flanked by Floquet boundary conditions describing the periodicity. Port conditions are used both for specifying the incident wave and for letting the resulting solution leave the model without any non-physical reflections. This simulation is equivalent to an infinitely extended periodic structure in the horizontal direction and therefore neglects any edge effects of the real meander^15^,^16^,^17^. The estimated accuracy of the simulations is 10^−3^, which is enough for the evaluation of the design and the comparison with the experimental results. All the simulations and measurements were performed for a 1550 nm wavelength. Figs. 1(a) and (b) show the electric field intensity for the TM wave and TE wave, respectively. The thickness *t* and width *w* of the superconducting NbN nanowire were selected to be 7 nm and 50 nm, respectively, with a pitch *p* of 100 nm, which are reasonable geometric values for fabrication. The refractive indices of NbN, Si and SiO_2_ were considered to be 5.23–5.82i, 3.45 and 1.45, respectively^15^,^16^. The thickness of SiO_2_ layer was a quarter of the wavelength. The energy of the electric field spills out of the nanowire in Fig. 1(a) because of the charges induced by the electrical field perpendicular to the nanowire. However, a relatively uniform electric field appears across the nanowire for the TE wave in Fig. 1(b). Given the field distribution, the absorptance (ABS) was determined by:
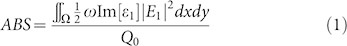
where *ω* is the angular frequency of the incident wave, *Q_0_* is the incident power, ω is the cross section of the nanowire, *ε_1_* is the permittivity of the superconducting nanowire and *E_1_* is the internal electric field of the nanowire. To more intuitively illustrate the absorptance, the electromagnetic power loss densities (absorptance) in the nanowire are presented in Figs. 1(c) and (d) for TM and TE waves, respectively, which indicate that the absorptance for the TM wave is effectively smaller than that of the TE wave. In other words, SNSPDs may exhibit strong polarization sensitivity.

**Figure 2:**
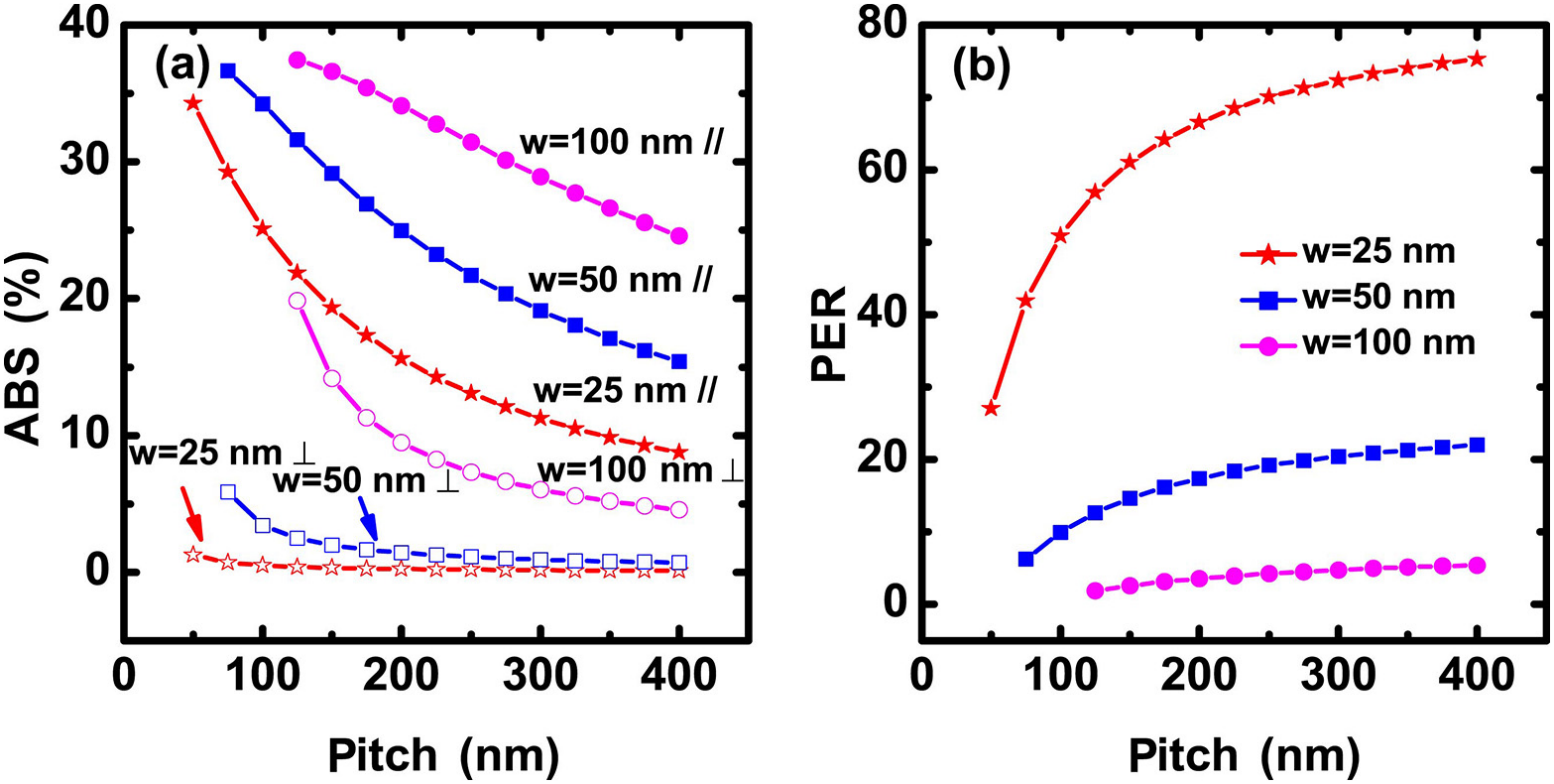
Calculation of ABS and PER. (a) Numerical results of the absorptance (ABS) of NbN nanowires with a thickness of 7 nm as a function of pitch for various widths, TE (//) and TM (⊥) waves. (b) Calculated PER versus pitch for various linewidths using the data in (a).

Fig. 2(a) shows the calculated absorptance dependence on the pitch for TM and TE waves. Three representative widths (25, 50, and 100 nm) of the nanowire were selected for comparison. A thickness of 7 nm was adopted for the calculation, which is a reasonable value for a high-performance SNSPD fabricated on a Si substrate. In principle, a high filling ratio (large width and small pitch) corresponds to a high absorptance for both TM and TE waves. However, the enhancement of absorptance for the TM wave is stronger than that for the TE wave. The corresponding polarization extinction ratio (PER) for the SNSPD is shown in Fig. 2(b). Here the PER is defined as the absorptance ratio of the TE to TM wave. Fig. 2(b) demonstrates that a smaller width and a larger pitch result in a larger PER. For example, the PER may reach 70 for a SNSPD with parameters of *w* = 25 nm and *p* = 250 nm. However, the corresponding maximal absorptance for the TE wave is only 12%. Therefore, there is a tradeoff for a SNSPD with a high PER and an acceptable DE. In addition, the process complexity of the device fabrication should also be considered.

### Experimental results

**Figure 3:**
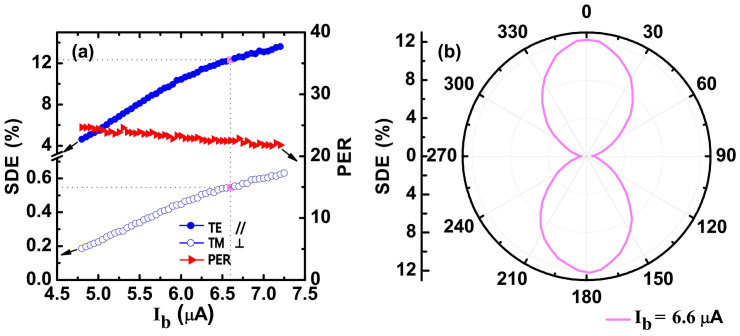
Measurement results of SNSPD. (a) SDE and PER as functions of bias current. The blue solid and hollow dots represent the SDE for TE and TM waves, respectively. The PER, represented by the red triangles, is calculated from the SDE data for two different polarizations. (b) The polarization dependence of SDE in the polar coordinate system at a dark count rate of 100 Hz.

SNSPD devices were fabricated from NbN film deposited on thermal oxidized Si substrate and packaged with light illuminated from the backside of the substrate. The devices were cooled down to 2.3 K for the characterization. Detailed information concerning device preparation, characterization and measurement setup can be found in the methods' section below. Fig. 3(a) shows the current dependence of the system detection efficiency (SDE) for parallel and vertical polarizations as well as the PER for a 50 nm-linewidth and 250 nm-pitch device. The SDE for the parallel polarization is one order of magnitude higher than the SDE for vertical polarization, which yields a PER range of 21–25 for the entire bias range. The slight decrease of PER in the bias range was unclear at this point. At the dark count rate of 100 Hz marked with pink dots, we obtained a PER of 22 with a maximal SDE of 12%. By tuning the state of polarization (SOP), the polarization dependence of the SDE was measured and is displayed in Fig. 3(b) when the device was biased at a current of 6.6 μA with a dark count rate of 100 Hz. The “peanut” curve further demonstrates the strong polarization sensitivity of the SNSPD.

**Figure 4:**
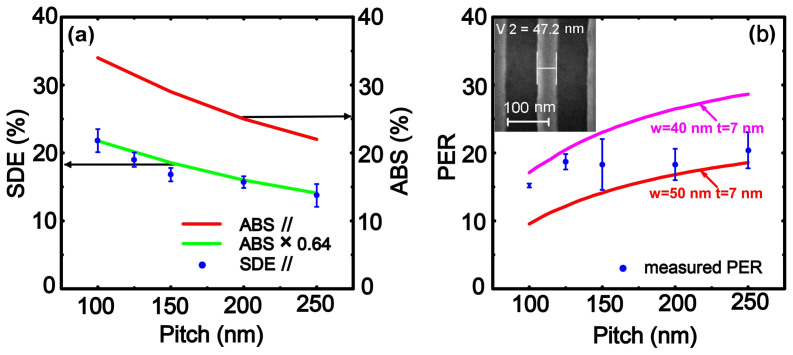
Comparison and analysis of 16 SNSPDs with different pitches. (a) Plot of SDE and ABS for the TE wave as a function of the pitch. The blue dots represent the measured SDEs of the devices. The error bar indicates the standard deviation of the SDEs for SNSPDs with the same pitch. The red line represents the calculated absorptance, and the green line shows the estimated SDE from the absorptance curve considering a loss factor of 0.64. (b) Comparison of the measured PERs (blue scattered dots) with the calculated PER as a function of pitch. The red and pink lines represent linewidths of 50 and 40 nm, respectively. The inset presents an SEM image of a typical nanowire, revealing a linewidth of 47.2 nm. The error bar indicates the standard deviation of the PERs for SNSPDs with the same pitch.

To study the pitch dependence of SDE and PER, 16 SNSPDs with 5 different pitch values (100, 125, 150, 200, and 250 nm) were measured. The blue dots in Fig. 4(a) represent the measured maximal SDEs of SNSPDs for TE wave with different pitches in the same measurement condition. The measured maximal SDEs are smaller than the calculated ABS for the TE wave represented by the red line. However, considering the existing system optical loss and non-unity intrinsic detection efficiency, a factor may be adopted to reexamine the relation between ABS and SDE. Represented by the green curve in Fig. 4(a), the measured SDE matches with the absorptance with a factor of 0.64. Furthermore, the PERs for SNSPDs with different pitches were calculated according to the measured SDE (//) and SDE (⊥) and are shown in Fig. 4(b), which deviate from the calculated values (red curves in Fig. 4(b)). This finding may be explained by the discrepancy of the width of the nanowire from the designed value, though other issues like the film thickness may also have contribution to it. The calculation results in Fig. 2(b) indicate that PER is very sensitive to the linewidth of the nanowire especially when the linewidth is smaller. One typical scanning electron microscopic (SEM) image of the nanowire shown as the inset of Fig. 4(b) reveals that the real linewidth may be slightly smaller than 50 nm. A smaller linewidth of 40 nm results in a higher PER, as represented by the pink line in Fig. 4(b). The measured PER values are almost all located between the two curves for the 50 nm and 40-nm widths.

## Discussion

We have demonstrated that an SNSPD with a PER over 20 and SDE of 12% can be fabricated. By improving the optical coupling and material optimization, the SDE can be further increased to the maximal absorptance (>20%). In future studies, the width of the nanowire should be decreased in order to increase the PER. However, there will be some process challenges such as those involved with the electron beam lithography. The use of the popular optical cavity structure may effectively improve the SDE by increasing ABS. However, the PER decreases because the absorptance for a TM wave increases much more than that for a TE wave. Therefore, it is necessary to develop a new polarization-selective optical structure to increase both the DE and PER in the future, like integrated plasmonic structures proposed by Csete et al.^18^,^19^.

In summary, an approach to produce a single photon detector with high polarization sensitivity was reported. By fabricating an SNSPD with a width of 50 nm and pitch of 250 nm, an SNSPD with a SDE of 12% and a PER of 22 was produced. The SDE and PER dependence on the width and pitch were systematically studied using both simulations and experiments, and the results match well with each other. We believe that there is much room for improving both PER and SDE. The SNSPD with high PER provides a unique solution for single photon counting applications based on polarization sensitivity, such as polarization imaging.

## Methods

### Device preparation

NbN thin film with a thickness of 7 nm was deposited on a double-side oxidized silicon substrate at room temperature using reactive DC magnetron sputtering in a mixture of Ar and N_2_ gases. The film was patterned into a meandered nanowire structure by electron beam lithography using a positive-tone Polymethyl Methacrylate electron-beam resist and was reactively etched in CF_4_ plasma. The nanowire covers an active area of 15 × 15 μm^2^ with a linewidth of 50 nm, which is easier to fabricate and control than a 25 nm-wide nanowire. Five different pitches (100, 125, 150, 200, and 250 nm) were selected for five different types of SNSPDs on the same wafer. Each wafer includes 25 devices and each type has 5 samples. A 50-Ω-matched coplanar transmission line was formed using ultraviolet lithography and reactive ion etching. A lensed optical fiber was vertically aligned to the sensitive area from the backside of the substrate using a copper package-box. The distance between the fiber tip and substrate was controlled around 20 ± 5 μm to ensure good optical focus. The beam waist of the focused light-spot is about 10 μm. Then, the SNSPDs were mounted in a custom-designed 6-channel cryostat based on a compact Gifford–McMahon cryocooler and cooled to 2.3 K. All the 25 detectors on the same chip with five different geometric parameters were measured. Due to the existence of possible defects or the imperfect fabrication process, 9 detectors had suppressed critical currents (<5.0 μA) and the results were discarded. All the other 16 SNSPDs had critical currents between 6.7 μA and 9.3 μA. The critical currents and the corresponding width/pitch of the 16 detectors are shown in Table 1. Indeed it is difficult to give the precise widths based on the SEM measurement, and the variation of the linewidth exists even for one nanowire. Besides, multiple measurement by SEM may damage the detector. The variation of I_c_ is related to the possible defects in or the different width/thickness of the nanowire. We discussed the data of the 16 SNSPDs and there are at least two detectors for each pitch.

### Measurement setup

**Figure 5:**
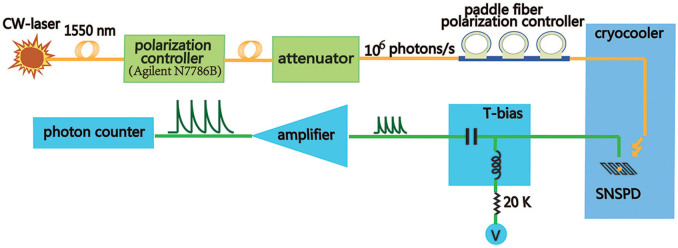
Schematics of the measurement system. Optical (represented yellow line) and electric (represented by green line) components are shown in the figure.

Schematics of the optical and electrical setups are shown in Fig. 5. A fiber-coupled continuous-wave tunable laser source (Agilent 81980A) was used as the photon source. A 2-port polarization controller (Agilent N7786B) was adopted to provide photons with any linear polarization. Due to the minimal power limitation (−10 dBm) of Agilent N7786B, three attenuators were adopted after N7786B, which provided the light intensity to a level of 10^6^ photons/s. Since the attenuators and the fibers connected may also change the SOP of the photons, a paddle fiber polarization controller was inserted to further adjust the SOP of photons arriving at the device. When the SOP of photons was optimized, the paddle fiber polarization controller was fixed. Then the SOP was precisely adjusted by Agilent N7786B. For the electrical characterization, an isolated voltage source in series with a 20 kω resister was adopted as a quasi-constant-current source for biasing. Voltage pulses arising from SNSPD were amplified using a 50 dB low noise amplifier (RF Bay Inc. LNA-650) and discriminated by a 200 MHz frequency photon counter (ThinkSRS SR400). All the electronics and optics were operated at room temperature except the fibers and coaxial cables were connected from room temperature to low temperature SNSPDs.

## Figures and Tables

**Table 1:** Parameters (critical current, width/pitch) of 16 detectors

Sample No.	Designed Width/Pitch (nm)	Measured Width/Pitch (nm)	I_c_ (μA)
1.	50/100	45.5/104	7
2.	50/100	45.0/102	7.1
3.	50/125	45.4/130	7.3
4.	50/125	47.3/129	6.8
5.	50/125	43.8/128	8.2
6.	50/150	42.6/152	7
7.	50/150	49.0/148	6.7
8.	50/200	55.0/201	7.6
9.	50/200	50.0/194	8.9
10.	50/200	42.2/195	9.1
11.	50/200	48.7/203	7.6
12.	50/200	52.0/203	8.9
13.	50/250	57.3/251	8.7
14.	50/250	47.5/241	9.3
15.	50/250	44.0/251	8.2
16.	50/250	50.3/253	7.8
